# Effectiveness of medication self-management, self-monitoring and a lifestyle intervention on hypertension in poorly controlled patients: The MEDICHY randomized trial

**DOI:** 10.3389/fcvm.2024.1355037

**Published:** 2024-05-21

**Authors:** Fabián Unda Villafuerte, Joan Llobera Cànaves, Andreu Estela Mantolan, Patricia Bassante Flores, Fernando Rigo Carratalà, Ana Requena Hernández, Bartolomé Oliver Oliver, Joan Pou Bordoy, María Lucía Moreno Sancho, Alfonso Leiva, Patricia Lorente Montalvo, The MEDICHY Group, Carmen Vega Martínez

**Affiliations:** ^1^ Sóller-Serra Nord Healthcare Center, Sóller, Spain; ^2^ Red de Investigación Cooperativa de Atención Primaria y Promoción de La Salud (RICAPPS)—Carlos III Health Institute (ISCIII), Madrid, Spain; ^3^ Balearic Islands Health Research Institute (IdISBa), Palma, Spain; ^4^ Primary Care Research Unit of Mallorca, Balearic Health Services (IB-Salut), Escola Graduada n3, Palma, Spain; ^5^ Dalt San Joan Healthcare Center, Mahó, Fornells, Maó, Spain; ^6^ Palliative Care Health Center, Palma, Spain; ^7^ San Agustín Healthcare Center, Palma, Spain; ^8^ Healthcare Basic-Unit Alqueria (Santanyí Healthcare Center), Santanyí, Spain; ^9^ Spanish Society of Family and Community Pharmacy, Madrid, Spain; ^10^ Camp Redo Healthcare Center, Palma, Spain; ^11^ Enfermería Familiar y Comunitaria, Programa Pacient Actiu de les Illes Balears, Gerencia de Atención Primaria de Mallorca, Escola Graduada n3, Palma, Spain; ^12^ Balearic Public Health Service (Ib-Salut), Palma, Spain

**Keywords:** blood pressure, hypertension, randomized trial, self-monitoring, primary care

## Abstract

**Background:**

Uncontrolled hypertension is a common problem worldwide, despite the availability of many effective antihypertensive drugs and lifestyle interventions. We assessed the efficacy of a multi-component intervention in individuals with uncontrolled hypertension in a primary care setting.

**Methods:**

This study was a randomized, multicenter, parallel, two-arm, single-blind controlled trial performed in primary healthcare centers in Mallorca (Spain). All participants were 35 to 75-years-old and had poorly controlled hypertension. Patients were randomly assigned in a 1:1 ratio to a control group (usual care) or an intervention group (self-monitoring of blood pressure, self-titration of hypertensive medications, dietary interventions, and physical activity interventions). The primary outcome was decrease in the mean SBP at 6 months relative to baseline.

**Results:**

A total of 153 participants were randomized to an intervention group (77) or a control group (76). After 6 months, the intervention group had a significantly lower systolic blood pressure (135.1 mmHg [±14.8] *vs.* 142.7 mmHg [±15.0], adjusted mean difference: 8.7 mmHg [95% CI: 3.4, 13.9], *p* < 0.001) and a significantly lower diastolic blood pressure (83.5 mmHg [±8.8] *vs.* 87.00 mmHg [±9.0], adjusted mean difference: 5.4 [95% CI: 2.9, 7.8], *p* < 0.0001). The intervention group also had significantly more patients who achieved successful blood pressure control (<140/90 mmHg; 54.4% *vs.* 32.9%, *p* = 0.011).

**Discussion:**

Self-monitoring of blood pressure in combination with self-management of hypertensive medications, diet, and physical activity in a primary care setting leads to significantly lower blood pressure in patients with poorly controlled hypertension.

**Clinical Trial Registration:** ClinicalTrials.gov, identifier ISRCTN14433778.

## Background

Hypertension is associated with increased morbidity and mortality and is the major modifiable risk factor for cardiovascular disease (1). At the population level, hypertension is responsible for significant temporary and permanent work disability and has a substantial negative economic impact (2). Hypertension is not successfully controlled in many individuals and is therefore a world-wide public health problem that is a challenge for health professionals at all levels of care.

Hypertension affects approximately one-third of the world's population who are between the ages of 30 and 79-years-old (3). The overall worldwide percentage of people with successful control of hypertension in 2019 was 23% for women and 18% for men, although control is generally less effective in low- and middle-income countries than in upper- and high-income countries. The successful control of hypertension in upper-income countries is 43% in women and 37% in men (4).

Tobacco consumption, alcohol abuse, excess dietary salt, sedentary lifestyle, and overweight are the main factors responsible for hypertension (5). For patients using anti-hypertensive drugs, therapeutic inertia (6), incorrect doses, inadequate combinations of drugs, and poor adherence to treatment (7) are responsible for the inadequate control of hypertension. Several interventional studies (8) have examined each of these factors in an effort to improve control of hypertension, but the results have been inconsistent.

To successfully control hypertension, it is necessary to implement established interventions that have known benefits in primary care settings or develop new interventions and then study their effectiveness. There are many reasons for the poor control of hypertension, and interventions should therefore target different these different causes, either individually or as multi-component interventions that consider these multiple causes.

The 2017 ACC/AHA guidelines (9) and the 2018 ESH/ESH guidelines use the GRADE system to classify the quality of evidence and evaluate the strength of recommendations for the non-pharmacological control of hypertension. The ACC/AHA guidelines assign grade 1A to losing weight for overweight people, consuming a heart-healthy diet [such as the Dietary Approaches to Stop Hypertension (DASH) diet], reducing dietary sodium, dietary supplements of potassium, increasing physical activity using a structured program, avoidance of alcohol, and cessation of smoking. The 2018 ESC and ESH guidelines assign grade 1A for losing weight and reducing sodium intake but assign category 1B to the other recommendations (10).

Clinicians in primary care centers diagnose, treat, and monitor most patients who present with hypertension and cardiovascular risk factors (3). However, very few clinical trials have examined the efficacy of these interventions in primary care settings. Instead, most research in this area has examined large hospital centers that are managed by local or state governments. An additional consideration is that self-measured blood pressure monitoring, which can provide clinicians with data for therapeutic decision-making and improve the patient's understanding of hypertension, may be an effective first step for the self-management of hypertension (11, 12).

In the context of hypertension, McManus et al. found that medication management based on self-monitoring of blood pressure with medication adjustment improved the control of blood pressure when used with or without telemonitoring (13, 14), and that this intervention was also effective for patients with high cardiovascular risk (15). Additionally, healthcare professional-led interventions targeting lifestyle modifications have demonstrated effectiveness in reducing hypertension (16). However, there is currently a lack of evidence supporting the effectiveness of a multicomponent intervention that incorporates self-monitoring, self-management of medication, and lifestyle interventions.

The objective of the present 6-month follow-up study of patients with uncontrolled hypertension was to test the efficacy of a multi-component intervention in a primary care setting that implements self-measured blood pressure monitoring, self-management of antihypertensive medications, the DASH diet, and increased physical activity on systolic blood pressure (SBP).

## Methods

### Study design and characteristics of patients

**Figure 1:**
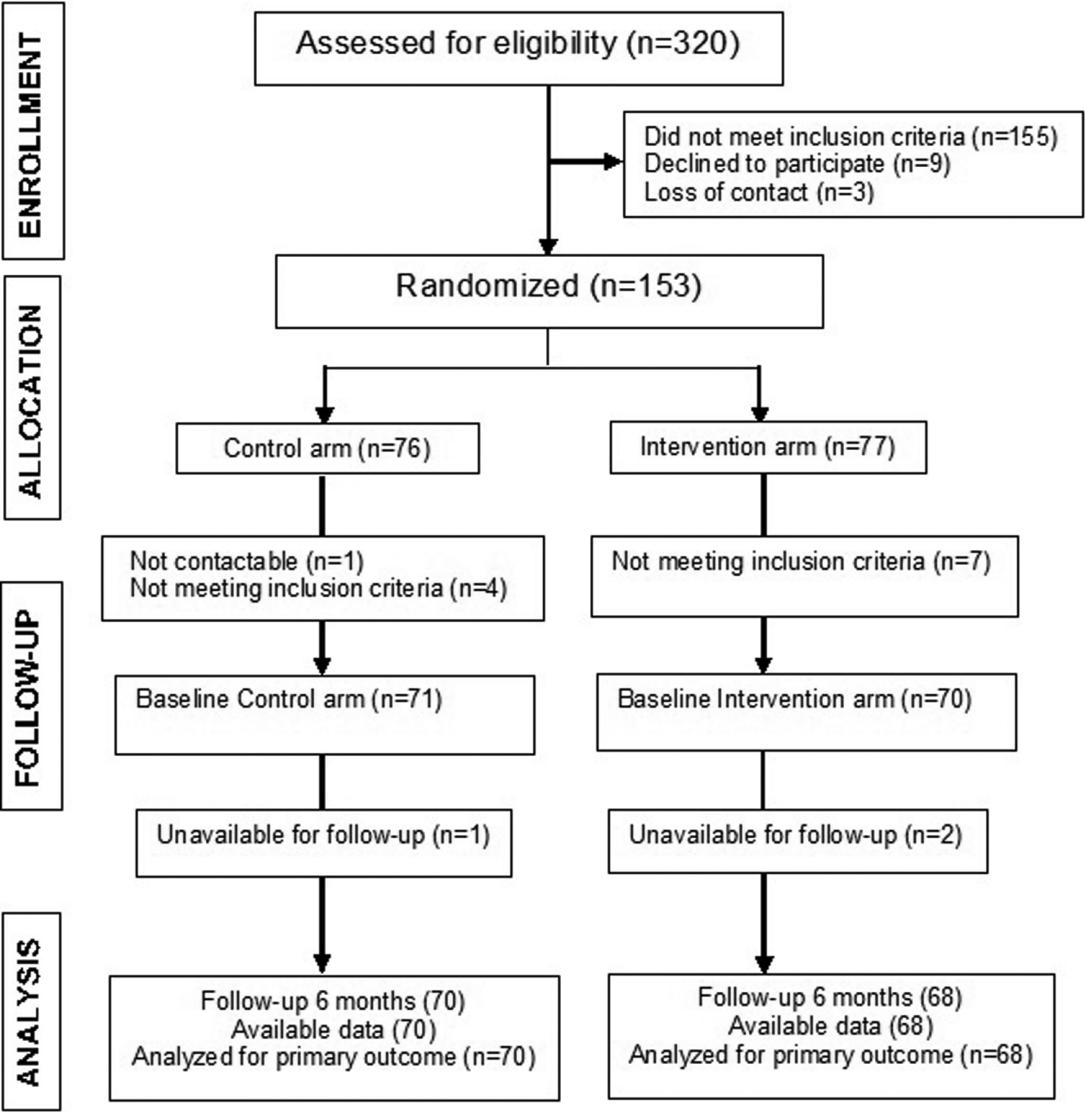
Study design and patient disposition (CONSORT flow diagram).

This randomized, multicenter, parallel, two-arm, single-blind, controlled trial was performed in seven primary health care centers (Sóller Serra-Nord, San Agustín, Escuela Graduada, Son Serra-La Vileta, Playa de Palma, Camp Redó, Son Pizà-Dra. Teresa Piqué) that were in two Healthcare Districts (Ponent and Migjorn) in Mallorca (Spain) and had 62 participating general practitioners (GPs). GPs from control patients were instructed to adhere to the ESC/ESH Guidelines for the management of arterial hypertension and provide brief counseling on dietary recommendations, salt intake, and physical exercise. The study was performed from January to December of 2022. The included patients were 35–75-years-old, used one or more antihypertensive medications, and had poorly controlled blood pressure (≥135/85 mmHg) based on a 3-day self-monitoring of blood pressure protocol as recorded in the electronic medical records (Figure 1). Individuals with a score of 12 or more on the Epworth Sleepiness Scale were excluded due to a high suspicion of obstructive sleep apnea (17).

### Exclusion criteria

The exclusion criteria were: (a) receipt of dialysis or diagnosis of renal failure; (b) myocardial infarction, heart bypass or angioplasty, stroke, peripheral arterial disease, heart failure, advanced liver disease, or atrial fibrillation; (c) inability or unwillingness to sign the consent agreement; (d) Alzheimer's disease, advanced dementia, or life expectancy less than the duration of the follow-up period (6 months); (e) indefinite institutionalization in a social health center; (f) prolonged treatment with a corticosteroid; (g) secondary hypertension; (h) impaired renal function based on a high urine albumin, values above 300 mg/24 h or a low estimated glomerular filtration rate (eGFR <60 ml/min/1.73 m2); (i) Epworth Sleepiness Scale >= 12 or obstructive sleep apnea.

This study was registered as a randomized controlled clinical trial (ISRCTN14433778), and a complete description was previously published (18). The conclusions drawn from the pilot study and the challenges encountered in recruiting and following up with patients during the COVID-19 pandemic, prompted several modifications to the initial study design. These adjustments involved enrolling patients with uncontrolled hypertension who were taking one or more medications, instead of those taking two or more. Furthermore, the final assessment was at 6 months instead of the 12 months initially planned, and the study design was altered to incorporate patient randomization.

### Randomization

All patients who signed the informed consent agreement were randomly assigned in a 1:1 ratio to the control group (usual care) or the intervention group (self-measured blood pressure monitoring, self-management of hypertensive medication, change of diet, and increasing physical activity). Randomization was performed using a central web-based system (OXMaR, www.ccmp.ox.ac.uk/oxmar). The researchers who performed the statistical analysis and the researchers who evaluated the main and secondary outcomes were separate from the research and recruiting team and were blinded to group allocations.

### Procedures

The researchers visited seven primary health care centers to provide detailed information about the study and ask for the assistance of 74 GPs, 62 of whom agreed. A computer search was performed to identify eligible patients, and they were then invited to participate by telephone. During this telephone call, each individual was given the Epworth Sleepiness Scale, and those with a score of 12 or more were excluded (17). Each included patient received an appointment at the primary health care centers. At the first visit (60 min), each patient was given an Omron M7 Intelli IT blood pressure monitor (HEM-7361T-EBK), a device approved by the ESH, ISH, and the World Hypertension League (www.stridebp.org). Each patient was also given a form and asked to record three blood pressure measurements in the morning and three measurements in the afternoon on three straight days, with an interval of 1–2 min between consecutive measurements. An on-site workshop was used to train all patients in the methods to be used for these measurements.

At the second visit (25 min), the records were collected, and blood pressure values were averaged, with exclusion of the first of the three readings from the morning and afternoon. A SBP of 135 mmHg or more or a diastolic blood pressure (DBP) of 85 mmHg or more was defined as uncontrolled hypertension. Then, the signed informed consent agreements were collected, and the patients were randomized.

At the third visit (45 min), a member of the external research team recorded 3 blood pressure readings; measured body weight, height, and abdominal circumference; administered a dietary survey (19) and the Spanish version of the quality of life survey (20); assessed physical activity using the International Physical Activity Questionnaire (IPAQ); and metabolic equivalents (MET)-minutes per week were determined by multiplying the MET intensity with the duration of each activity over the course of seven days (21). The patients in the intervention group were given an appointment with a team of nurses who were trained in the administration of the intervention. These nurses explained the procedures and administered necessary materials as the beginning of the monthly follow-up.

### Intervention

The intervention consisted of self-measured blood pressure monitoring, self-management of antihypertensive medication, and promotion of a healthy lifestyle.

#### Blood pressure measurements

Each patient received a blood pressure monitor, a chart showing the procedures for making these measurements, and a form to record the measurements. Blood pressure was recorded 2 times in the morning and 2 times in the afternoon during the first 5 days of each month, for six months (11). The nurse reviewed the use of the monitor and adjusted the cuff so that it fit the upper arm of each patient.

#### Optimization of treatment and self-management of medication

Treatment optimization and self-management consisted of one or two steps that intensified the pharmacological treatment according to the 2018 European Hypertension Guidelines (10) — increasing the dose of the current antihypertensive agent— when there were 5 blood pressure readings above 150/95 mmHg [the threshold recommended in the 2023 European guidelines (22) for pharmacological treatment in all cases], the patient applied the recommendation of pharmacological intensification. A new antihypertensive agent or a reduction in the pharmacological treatment (when five blood pressure readings were 100/60 mmHg or lower) was initiated through a telephone call from the doctor to the patient, following an appointment arranged by the study nurse. Each month, the intervention nurses collected the self-monitored blood pressure results and recalled the components of the intervention.

#### Changes in diet

The intervention nurses administered materials and instructions for implementation of the DASH diet individually or in groups of up to 4 patients. The purpose of this diet is to reduce blood pressure and the risk of cardiovascular disease by emphasizing foods that are high in calcium, potassium, magnesium, and fiber, and low in sodium (23, 24). This diet does not require special foods, but instead sets daily nutritional goals based on servings from different food groups. More specifically, this plan recommends eating vegetables, fruits, whole grains, fat-free or low-fat dairy products, fish, poultry, legumes, nuts, and vegetable oils. Patients also learned to read food product labels and were asked to avoid alcoholic drinks.

#### Physical exercise

The intervention nurses prescribed intensification of physical activity chosen in the study, walking, by considering the patient's initial physical condition and delivering individualized plans that consisted of increasing the duration, distance, and/or speed. First, the risks associated with increased physical activity were evaluated, and progressive training was prescribed, with self-control by the patients according to their preferences and using measurements of heart rate, distance, steps, time, or a Borg Index (25) between levels 12 and 13 (moderate perceived exertion). Patients eligible for physical activity were stratified into three groups based on their initial level of physical activity: those with a weekly activity of less than 600 METs (Group A), between 600 and 900 METs (Group B), and over 900 METs (Group C). Each group starts at different intensity levels, Group A patients commenced with walking for 10–12 min, three days a week, during the first two weeks. Group B patients engaged in walking for 25 min, four to five days a week, while Group C patients walked for 50 min, five to seven days a week. Following this initial period, the intensity of activity was progressively increased.

The materials that described the exercise intervention were delivered to the patients in print and were also available on a web site. These materials included information that were related to each aspect of the intervention, such as healthy route maps, self-monitoring of blood pressure, and physical activity videos. The professionals had access to pharmacological optimization videos and the main guidelines. A blog was also established to encourage open communications and links to social networks (https://automanejodelahipertension.wordpress.com).

At the end of the intervention period, researchers in the external unit recorded three consecutive blood pressure values, anthropometric data, and re-administered the surveys that were given at the beginning of the study for all patients in both groups.

### Primary outcome

The primary outcome was a significant decrease in the mean SBP at 6 months relative to baseline.

Blood pressure was measured after 5 min of rest using a validated electronic automated sphygmomanometer (OMROM 7 Intelli IT). These measurements were performed while the patient was seated, with the back supported and the feet on the floor, without speaking, and with no eating or smoking during the previous 30 min (10). Three blood pressure readings were recorded at 1 min intervals using a suitable cuff size and with the arm resting on a table. The mean of the second and third readings was calculated and recorded.

Subgroup analysis was also used to assess the effectiveness of the intervention in reducing SBP at 6 months according to gender, low or moderate cardiovascular risk, BMI above or below 30, and presence or absence of diabetes.

### Secondary outcomes

The secondary outcomes were achieving adequate SBP and DBP values (<140/90 mmHg) after 6 months; an improvement in quality of life, as measured by a validated Spanish language version of the EuroQol-5D (20); adherence to the antihypertensive medication regimen, based on the registry in the prescription section of the electronic medical record of the patient (Medication Possession Ratio) (26);decreased body mass index (BMI) and increased physical activity, as determined by the International Physical activity Questionnaire (IPAQ) (21); Adherence to the DASH diet was assessed using a comprehensive food frequency questionnaire consisting of 41 items. The DASH Adequacy Index (DASH-AI) was computed to evaluate adherence to the DASH dietary pattern, which includes the following criteria: total fat (≤ 27% of total energy per day), saturated fatty acids (≤ 6% of total energy per day), proteins (≥ 18% of total energy per day), sodium (< 2,300 mg/day), potassium (≥ 4,700 mg/day), calcium (≥ 1,250 mg/day), magnesium (≥ 500 mg/day), fiber (≥ 30 g/day), cholesterol (≤ 27 mg/day), and carbohydrates (≤ 55% of total energy per day) and patient safety (hypertensive crises, number of hypotensive episodes that required an emergency department visit, and adverse effects associated with medications).

### Statistical analysis

The necessary sample size was calculated as at least 177 patients per group, assuming a clinically relevant difference of SBP (5 mmHg) between the groups, with 80% power, a two-sided α value of 0.05, and an estimated a standard deviation (SD) of 17 mmHg (13).

For the primary outcome, an unadjusted analysis was used to assess the effectiveness of the intervention using Student's *t*-test; an adjusted analysis used a generalized linear model (GLM) with a normal distribution and an identity link function, with adjustment for baseline SBP. For the secondary outcomes, a GLM was also used for analysis. To analyze the effectiveness of the intervention on DBP, treatment adherence, and BMI, and body weight, a GLM with a normal distribution and an identity link function was used; and to analyze improvements in blood pressure control, quality of life and physical activity, a GLM with a binomial distribution and a logit link function was used. The number of changes in the antihypertensive medication regimen was also analyzed using a GLM with a Poisson distribution and log-link function.

All analyses, including subgroup analyses, were pre-specified in the initial protocol. In the subgroup analysis, a test for interaction in the adjusted models was used to evaluate statistically significant differences of subgroups.

All statistical analyses were performed using IBM SPSS Statistics (version 23; IBM Corp., Armonk, NY, USA) with a predefined analysis plan. All results were reported in accordance with the Consolidated Standards of Reporting Trials guidelines (S1 CONSORT Checklist).

## Results

We screened 320 candidates for eligibility (Figure 1). Eligible individuals had a blood pressure of at least 140/90 mmHg registered in the electronic health records during the previous 12 months and also had a blood pressure of this level or higher during the initial consultation. One patient in the control group was lost before the baseline visit. We randomly allocated 76 patients to the control group and 77 to the intervention group, four patients in the control group and seven in the intervention group was excluded after randomization because of failure to meet the inclusion criteria.

A total of 141 patients met the inclusion criteria and agreed to participate (Table 1). Most patients were 55–70-years-old, half of them had a BMI greater than 30 kg/m^2^, and they used a median of 2 antihypertensive drugs (angiotensin-converting enzyme inhibitors: 40.0%, diuretic: 22.6%, angiotensin II receptor blocker: 15.7%, calcium channel blockers: 13.6%, beta-blockers: 7,2%). One patient in the control group and two patients the intervention group were lost to follow-up.

**Table 1:** Baseline demographic and clinical characteristics of the control and intervention groups.

	Control^a^	Intervention^a^
Age, years	60.7 ± 8.9	59.9 ± 9.0
Male	35/71 (49.3)	32/70 (45.7)
Level of education		
None	2/60 (3.3)	2/63 (3.2)
Primary education	28/60 (46.7)	26/63 (41.3)
Secondary education	13/60 (21.7)	26/63 (41.3)
University studies	17/60 (28.3)	9/63 (14.3)
SBP, mmHg	142.9 ± 14.6	146.7 ± 13.9
DBP, mmHg	86.31 ± 9.0	89.38 ± 8.3
Severe hypertension (>=180/120 mmHg)	0/71 (0)	2/70 (2.86)
Hypertension duration, years	9.7 ± 6.3	7.5 ± 6.0
Number of AHTs	1.8 ± 0.9	1.7 ± 0.9
Adherence to AHT treatment	74.7 ± 35.2	76.55 ± 31.8
Physical activity		
Low	20/70 (28.6)	8/70 (11.4)
Moderate	41/70 (58.6)	53/70 (75.7)
High	9/70 (12.9)	9/70 (12.9)
Cardiovascular risk-factors		
Cigarette smoking	16/63 (25.4)	22/67 (32.8)
Dyslipidemia	42/70 (60.0)	43/70 (61.4)
Diabetes mellitus	12/71 (16.9)	10/70 (14.3)
Obesity (BMI >30 kg/m^2^)	38/69 (55.1)	35/68 (51.5)
Abdominal circumference (cm)	107.43 (12.3)	107.82 (13.9)
Glomerular filtration rate, ml/min/1.73 m^2^	** **	** **
Normal (>90)	28/64 (43.7)	35/66 (53.0)
Mild reduction (60–90)	36/64 (56.2)	31/66 (47.0)
Serum creatinine, mg/dl	0.83 (0.1)	0.82 (0.2)
Time of follow-up,6 months	8.36 (1.8)	8.76 (1.9)
REGICOR cardiovascular risk	5.98 (3.2)	6.28 (3.3)
EQ-5D		
Mobility (some problems/confined to bed)	9/67 (13.4)	14/70 (20.0)
Self-care (some problems/unable to wash)	1/68 (1.5)	0/70 (0)
Usual activities (no problems/some problems)	2/68 (2.9)	2/68 (2.9)
Pain/discomfort (moderate pain/extreme pain)	38/68 (55.9)	42/69 (60.9)
Anxiety/depression (moderate /extremely anxious)	33/68 (48.5)	32/70 (45.7)
EQ-VAS	71.86 (15.2)	69.31 (18.3)

BMI, body mass index; CHD, cardiovascular disease; SBP, systolic blood pressure; DBP, diastolic blood pressure; AHT, antihypertensive; EQ-VAS, EuroQol visual analogue scale; REGICOR, Framingham equation adjusted for the Spanish population; EQ-5D, EuroQol Group 5-dimensional scale.

^a^ Data indicate mean ± SD or *n*/*N* (%).

Comparison of the two groups at baseline indicated they were similar in most socio-demographic and clinical characteristics. However, the intervention group had more women, more patients who were physically active, and fewer patients who had type II diabetes (Table 1).

### Primary outcome

The primary analysis plan specified use of intention-to-treat analysis, but because very few patients were lost to follow-up, a per-protocol analysis was used for simplicity. After 6 months, the reduction in SBP was significantly greater in the intervention group (*p* < 0.0001; Table 2) and the mean difference in the adjusted analysis was 8.7 mmHg (95% CI: 3.4, 13.9). The average SBP decreased by 11.9 mmHg (±17.0) in the intervention group and decreased by 0.2 mmHg (±15.4) in the control group. Subgroup analyses showed that sex, age, cardiovascular risk factors, and diabetes had no significant effect on this relationship (Supplementary Material S2 Figure 2: Subgroup analysis).

**Table 2:** Primary outcome in the control and intervention groups at 6 months.

	Unadjusted analysis mean (SD)		*p*-value	Adjusted analysis^a^ mean difference (95% CI)		*p*-value	Adjusted analysis^b^ mean difference (95% CI)		*p*-value
	Control	Intervention		Control	Intervention		Control	Intervention	
Final SBP, mmHg	142.7 (15.0)	135.1 (14.8)	0.003	Ref.	9.3 (4.7,13.5)	<0.0001	Ref.	8.7 (3.4,13.9)	0.001
ΔSBP, mmHg	−0.2 (15.4)	−12.0 (17.0)	<0.0001						

SBP, systolic blood pressure; SD, standard deviation; ref, reference.

^a^ Adjusted for baseline SBP.

^b^ Adjusted for baseline SBP, sex, level of education, diabetes mellitus, physical activity and cigarette smoking.

### Secondary outcomes

The two groups also had a statistically significant difference in DBP at 6 months (*p* < 0.0001; Table 3), and the mean difference was 5.4 mmHg (95% CI: 2.9, 7.8) in the adjusted analysis. The average DBP decreased by 5.9 mmHg (±8.2) in the intervention group and increased by 0.7 mmHg (±7.8) in the control group.

**Table 3:** Secondary outcomes: DBP and euroQol-5D in the control and intervention groups at 6 months.

	Unadjusted analysis mean (SD)		*p*-value	Adjusted analysis^a^ mean difference (95% CI)		*p*-value
	Control	Intervention		Control	Intervention	
Final DBP, mmHg	87.0 (9.0)	83.5 (8.8)	0.022	Ref.	5.4 (2.9;7.8)	<0.0001
ΔDBP, mmHg	0.7 (7.8)	−5.9 (8.2)	<0.0001			
	*n*/*N* (%)		* *	OR (95% CI)		
Blood pressure control (140/90 mmHg)	23/70 (32.9)	37/68 (54.4)	0.011	Ref.	2.4 (1.2;4.9)	0.011
EQ-5D						
Mobility (some problems/confined to bed)	10/67 (14.9)	14/66 (21.2)	0.346	Ref.	1.4 (0.5;3.4)	0.489
Self-care (some problems/unable to wash)	1/68 (1.5)	2/66 (3.0)	0.542	Ref.	2.1 (0.2;23.7)	0.550
Usual activities (no problems/some problems)	2/68 (2.9)	3/66 (4.5)	0.624	Ref.	1.6 (0.2;10.2)	0.623
Pain/discomfort (moderate pain/extreme pain)	38/68 (55.9)	42/67 (62.7)	0.421	Ref.	1.3 (0.6;2.9)	0.469
Anxiety/depression (moderate /extremely anxious)	30/68 (44.1)	36/67 (46.3)	0.802	Ref.	1.2 (0.5;2.5)	0.671
	Mean (SD)			Mean difference (95% CI)		
EQ-VAS	70.0 (17.6)	73.9 (16.1)	0.176	Ref.	4.77 (0.2;9.3)	0.041

SBP, systolic blood pressure; DBP, diastolic blood pressure; SD, standard deviation; Ref, reference; VAS: visual analogue scale.

^a^ Adjusted for baseline DBP and EQ-5D.

Table 3 also presents the percentage of patients who achieved blood pressure control, defined as a level of 140/90 mmHg or less. More patients in the intervention group than in the control group successfully attained this target (54.4% vs. 32.9%). The absolute risk reduction, ARR was 21.5% and the corresponding number needed to treat (NNT) for one patient to achieve the target BP was 4.6 (95% CI: 3.1, 10.9).

At the 6-month follow-up, the quality of life, measured by the EQ-5D, was not significantly different for the two study arms. However, the EQ-Visual Analogue Scale (VAS) had a greater improvement in the intervention group (mean difference = 4.8, 95% CI: 0.2, 9.3, *p* = 0.041).

Also, at the 6-month follow-up, there were four treatment-related adverse reactions, all in the intervention group; two patients experienced dizziness, patient one had pruritus, and one patient developed edema.

Changes in the antihypertensive medication regimen were similar in the two groups (Table 4). In addition, the two groups also had no significant differences in pharmacological adherence, DASH diet adherence, weight change, or physical activity.

**Table 4:** Secondary outcomes: adherence to the antihypertensive regimen, changes in HTA treatment, adherence to the DASH diet, body weight, BMI, and physical activity in the control and intervention groups at 6 months.

	Unadjusted analysis mean (SD)		*p*-value	Adjusted analysis^a^ mean difference (95% CI)		*p*-value
	Control	Intervention		Control	Intervention	
Adherence (%)	78.9 (3.0)	77.6 (3.0)	0.839	Ref.	0.4 (−0.4;1.2)	0.373
Number of HTA changes medication	0.3 (0.7)	0.4 (0.8)	0.300	Ref.	0.1 (−0.1;0.4)	0.308
Adherence to Dash Diet	2,0 (0,6)	2,0 (0.6)	0.428	Ref.	0.04 (0.22;0.14)	0.660
Weight	83.5 (15.7)	82.6 (20.3)	0.322	Ref.	−0.9 (−6.9;5.1)	0.767
BMI	30.6 (4.90)	30.0 (6.51)	0.289	Ref.	−0.6 (−2.5;1.4)	0.564
Physical activity	*n*/*N* (%)			OR (95% CI)		
Low	22/70 (31.4)	18/68 (26.5)		Ref.		
Moderate	46/70 (65.7)	42/68 (61.8)			0.3 (0.1–0.8)	0.021
High	2/70 (2.9)	8/68 (11.8)	0.125		12.9 (3.7–44.9)	0.001
HBP changes medication (%)	14/70 (20.0)	19/69 (27.5)	0.296	Ref.	1.5 (0.7;3.3)	0.298

SD, standard deviation; MD, mean differences, BMI, body mass index; HBP, high blood pressure.

^a^ Adjusted for baseline Adherence, number of HTA medication, adherence to dash diet, BMI and physical activity.

## Discussion

The present study of patients with uncontrolled hypertension in a primary care setting showed that an intervention consisting of self-measured blood pressure monitoring, a lifestyle intervention, and clear and precise indications for self-adjustment of antihypertensive drugs led to decreases of SBP and DBP after 6 months. Moreover, our subgroup analysis showed that sex, age, cardiovascular risk factors, and diabetes had no significant effect on this outcome.

Our results are also in line with those reported by Margolis et al. (27), who performed a randomized clinical trial that compared the effectiveness of a telemonitoring lifestyle intervention with usual care for the control of blood pressure. This previous study was performed in a primary care setting and reported a reduction of 11.3 mmHg in the SBP of the intervention group relative to the control group. These authors attributed this effect to the increased intensity of treatment (24%) and the increased use of home blood pressure monitoring (19%) (28). In the present MEDICHY study, the unadjusted difference in SBP between groups was of 11,7 mmHg. The mean number of changes in antihypertensive agents was also greater in the intervention group (0.44 vs. 0.31), although this difference was not statistically significant. The TASMINH2 clinical trial (13) also reported a benefit from self-monitoring of blood pressure, self-administration of antihypertensive drugs, and telemonitoring of home blood pressure measurements. Our study and these previous studies examined the effect of self-monitoring of blood pressure when performed in primary care settings, but these studies were performed in different health care systems. Our results are quite similar to those from the TASMINH2 study, which reported a mean decrease of SBP by 12.9 mmHg (95% CI: 10.4, 15.5) after 6 months in the self-management group; however, the TASMINH2 study also reported a decrease in the SBP in the control group, but our control group had no significant change in SBP.

The previous TASMINH2 study (13) randomized patients with hypertension to one of three arms: (a) treatment adjustment and self-monitoring, (b) treatment adjustment, self-monitoring, and telemonitoring, and (c) treatment adjustment (based only on clinical measurements) and usual care. These researchers reported a statistically significant reduction in blood pressure at 12 months in both intervention groups relative to the usual care group, and no significant difference between the two intervention groups. Therefore, telemonitoring appeared to provide no additional benefit. These results are consistent with our results. Tucker et al. (29) performed a meta-analysis in 2017 that analyzed the effect of self-monitoring on the reduction of blood pressure and concluded that did not decrease blood pressure but led to a clinically significant decrease in blood pressure for at least 12 months when it was combined with other interventions. They recommended use of self-monitoring of blood pressure with additional interventions that provide personalized support, such as changes in lifestyle or in adjustment of medications. The present MEDICHY study proposed personalized interventions that include increased physical activity, a low-sodium and low-calorie diet, SMBP, and guided adjustment of antihypertensive medications. The meta-analysis of Tucker et al. also found no differences in efficacy according to gender and most comorbidities, similar to the present study.

A 2008 study by Green et al. (30) examined whether a new model of care that used web-based patient services, self-measured blood pressure monitoring, and assistance from a pharmacist was effective in controlling blood pressure. Relative to their usual care group, their intervention group had significantly more patients who achieved blood pressure control (56% vs. 31%). These results are consistent with the results of the present study (54.4% vs. 32.9%). In addition, our study and the study of Green et al. were also similar in that the interventions consisted of self-monitoring, increased physical activity, and a pharmacological optimization plan. We also used a web page (but only for informational purposes) and a pharmaceutical professional made contributions to the adjustment of medications, although our interventions were implemented by a group of nurses.

A meta-review by Shahaj et al. (31) assessed the effectiveness of self-measured blood pressure monitoring and also identified several effective interventions. Providing patients with information on hypertension and treatment, self-monitoring, and telematic media are common and effective interventions for the control blood pressure. Self-monitoring by a patient who has a supportive relationship with a medical professional can increase the patient's perception of the importance of symptoms and foster confidence in the patient's self-management of hypertension. self-monitoring, with or without telehealth, can help to bridge the gap between a medical professional and a patient. The present MEDICHY study highlights the value of self-monitoring of blood pressure when used with other interventions that have the potential to help control of blood pressure. It is necessary to identify the effectiveness of these interventions in clinical trials and in real world studies that resemble routine clinical practice.

A limitation of the present study is the relatively short follow-up period of 6 months, which may have constrained our ability to assess the long-term effectiveness of the intervention, potentially leading to a diminishing impact over time. This duration appeared adequate for measuring changes in blood pressure, but not for assessing the effect of changes in lifestyle (diet and exercise). In fact, our two groups had no significant differences in BMI, quality of life, or adherence to antihypertensive treatment at 6 months. The number of participating patients was sufficient to identify significant differences of blood pressure between our two groups, but a larger number of patients may be needed to identify differences in these other outcome measures. Our intervention group had an increase in the quality of life over time, but the difference between the two groups was not significant, as also reported in the TASMINH2 study. This result could be due to our relatively short follow-up time and limited number of participants.

Adherence to behavioral and lifestyle changes can be difficult and time consuming for patients. For example, a publication in *The Lancet* that used data from the National Health and Nutrition Survey (NHANES) and was published on the 20th anniversary of the publication of the DASH diet, showed that less than 1% of the U.S. population was fully compliant this diet (32). In addition, it is difficult for individuals to reliably measure food portions when adopting the DASH diet. It is possible that the specific dietary alterations should be adapted to our local population. Another limitation of our study is that it was performed during the COVID-19 pandemic. Although this was unavoidable, it was obviously difficult to implement a field study with primary care GPs who also worked on the frontline of this pandemic; it was also difficult to encourage physical activity in patients who are convalescent. To increase the external validity of our findings, we suggest that similar studies be performed in subjects drawn from different populations by other investigators. This may increase the generalizability of our findings and possibly lead to the identification of more effective interventions.

This study was not designed to detect differences in cardiovascular outcomes. However, due to the significant difference in the SBP of the control and intervention groups, we expect that our intervention would decrease the risk of stroke, coronary heart disease. A 2021 meta-analysis (33) concluded that a 5 mm Hg reduction in SBP decreased the risk of serious cardiovascular events by approximately 10%, regardless of previous diagnoses of cardiovascular disease, even in patients with normal or high-normal blood pressure. This study also reported decreases in the risk of stroke (13%), heart failure (13%), ischemic heart disease (8%), and death from cardiovascular disease (5%). These benefits should be greater for patients with a greater cardiovascular risk at baseline. Another meta-analysis demonstrated a 34% decrease in the combined risk of stroke and coronary ischemia when the SBP decreases by 7 mmHg (34). Notably, our intervention led to a decrease of 11.4 mmHg in SBP at 6 months.

## Conclusions

An intervention in primary care, which included self-monitoring of blood pressure, a lifestyle intervention, and explicit and accurate guidance for self-adjusting antihypertensive medications, resulted in significant reductions in blood pressure after six months.

However, there were no differences in lifestyle behaviors between intervention and control patients. Self-management and self-monitoring is important in hypertension control and is also an essential element that is needed for the successful management of others chronic pathologies. Therefore, a commitment by the patient and the healthcare team is therefore necessary.

## Data Availability

The datasets presented in this study can be found in online repositories. The names of the repository/repositories and accession number(s) can be found below: https://zenodo.org/records/8337429.
