# Supplementary figures and images for: Effectiveness of medication self-management, self-monitoring and a lifestyle intervention on hypertension in poorly controlled patients: The MEDICHY randomized trial
Source: Front Cardiovasc Med. 2024 May 21;11:1355037. doi: 10.3389/fcvm.2024.1355037 (PMC11148777; doi:10.3389/fcvm.2024.1355037)

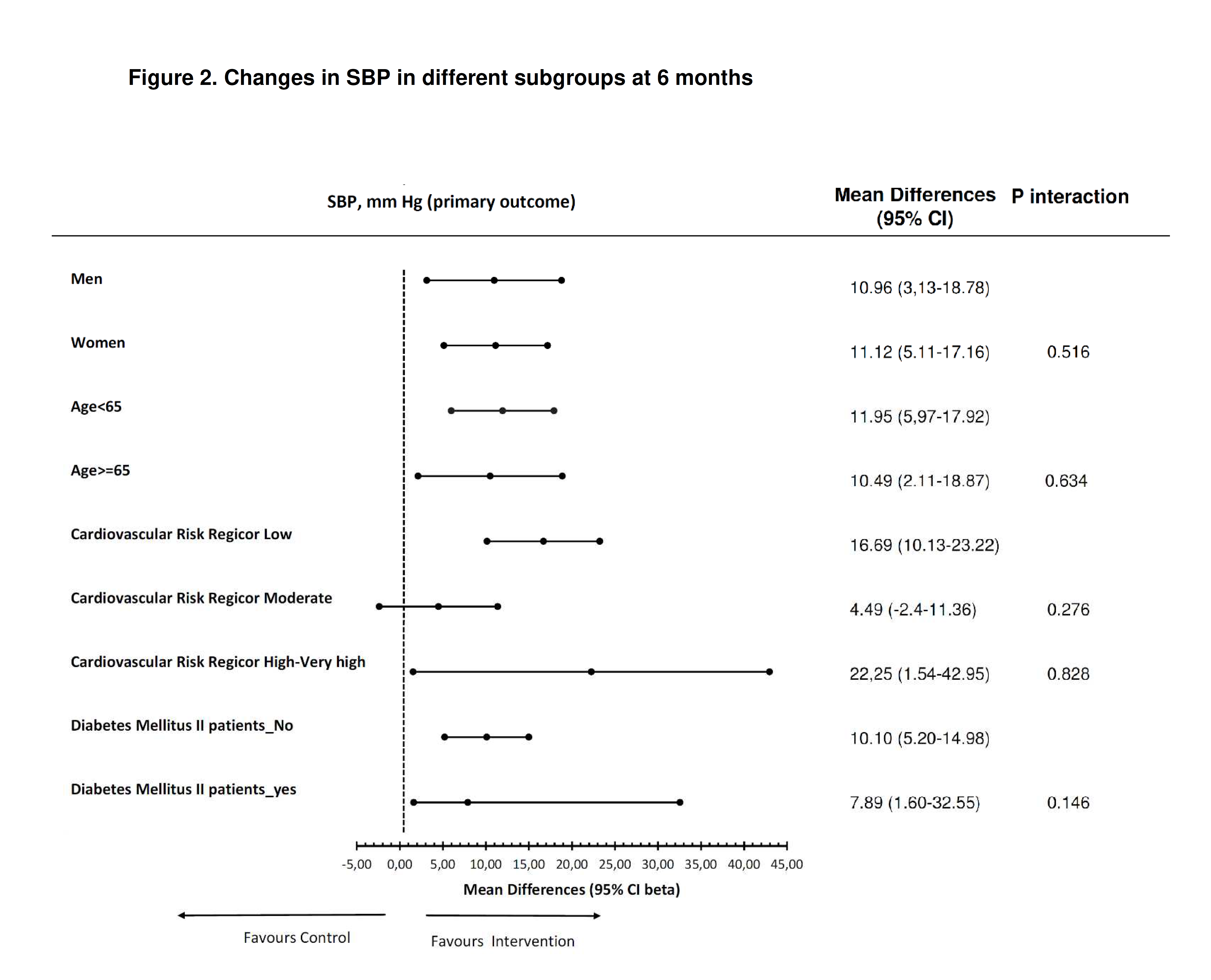

Supplement: Supplementary file 1 [file Image1.tif]
